# The clinical efficacy of a novel smartphone-based salivary self-test for the prediction of pre-eclampsia, pregnancy-induced hypertension and intrauterine growth restriction: a prospective cohort study

**DOI:** 10.3389/fmed.2024.1385299

**Published:** 2024-12-20

**Authors:** Ida Catharina Püschl, Lisbeth Bonde, Thomas Alexander Gerds, Mia Sato Tackney, James Quest, Bjarke Lund Sorensen, Nicholas Stephen Macklon

**Affiliations:** ^1^Faculty of Medicine, University of Copenhagen, Copenhagen, Denmark; ^2^Department of Obstetrics and Gynecology and ReproHealth Consortium, Zealand University Hospital, Roskilde, Denmark; ^3^Department of Gynecology, Juliane Marie Centre, Rigshospitalet, Copenhagen University Hospital, Copenhagen, Denmark; ^4^Department of Biostatistics, University of Copenhagen, Copenhagen, Denmark; ^5^Medical Research Center (MRC)-Biostatistics Unit, University of Cambridge, Cambridge, United Kingdom; ^6^Morgan Innovation and Technology, Petersfield, United Kingdom; ^7^London Women’s Clinic, London, United Kingdom

**Keywords:** pre-eclampsia, intrauterine growth restriction, fetal growth restriction, salivary uric acid, pregnancy-induced hypertension, gestational hypertension, placental health

## Abstract

**Introduction:**

This study investigated the efficacy of a digital health solution utilizing smartphone images of colorimetric test-strips for home-based salivary uric acid (sUA) measurement to predict pre-eclampsia (PE), pregnancy-induced hypertension (PIH), and intrauterine growth restriction (IUGR).

**Methods:**

495 pregnant women were included prospectively at Zealand University Hospital, Denmark. They performed weekly self-tests from mid-pregnancy until delivery and referred these for analysis by a smartphone-app. Baseline characteristics were obtained at recruitment and pregnancy outcomes from the journals. The mean compliance rate of self-testing was assessed. For the statistical analyses, standard color analyses deduced the images into the red-green-blue (RGB) color model value, to observe the individual, longitudinal pattern throughout the pregnancy for each outcome. Extended color analyses were applied, deducing the images into 72 individual color variables that reflected the four dominant color models. The individual discriminatory ability was assessed by calculating the area under the curve for the outcome of PE, and the outcome of hypertensive pregnancy disorders solely or combined with IUGR at 25 weeks of gestation and for the weekly color change between 20 and 25 weeks of gestation.

**Results:**

Thirty-four women (6.9%) developed PE, 17 (3.4%) PIH, and 10 (2.0%) IUGR. The overall mean compliance rate was 67%, increasing to 77% after updating the smartphone-app halfway through the study. The longitudinal pattern of the RGB value showed a wide within-person variability, and discrimination was not achieved. However, it was noted that all women with IUGR repeatedly had RGB values below 110, contrasting women with non-IUGR. Significant discriminatory ability was achieved for 8.2% of the analyses of individual color variables, of which 27.4% summarized the Hue color variable. However, the analyses lacked consistency regarding outcome group and gestational age.

**Conclusion:**

This study is the first proof-of-concept that digital self-tests utilizing colorimetric sUA measurement for the prediction of PE, PIH, and IUGR is acceptable to pregnant women. The discriminatory ability was not found be sufficient to have clinical value. However, being the first study that compares individual color variables of the four dominant color models, this study adds important methodological insights into the expanding field of smartphone-assisted colorimetric test-strips.

## Introduction

Hypertensive disorders of pregnancy and intrauterine growth restriction (IUGR) are common complications to pregnancy, affecting 2–8% (1) and 3–7% (2) of pregnancies, respectively. Pre-eclampsia (PE) and IUGR are associated with severe maternal and fetal morbidity, are among the leading causes of maternal and perinatal death (1, 3), and account for a significant healthcare cost burden (4). PE is defined as *de novo* onset of hypertension after 20 weeks of gestation in combination with either IUGR, proteinuria, or maternal organ dysfunction (5), while IUGR is a pathological restriction of the fetal genetic growth potential (3). Pre-eclampsia is generally accepted as a multifactorial syndrome with heterogeneous presentation and evolvement (6), making early identification of pregnancies at risk important (1). Several strategies designed to triage women into high and low risk have been proposed, leading to the integration of a first trimester screening-algorithm for early-onset PE (7–9). However, it lacks predictive value for overall-PE (8) and is unsuitable to low-resource settings (7).

Since IUGR has a shared etiology with PE, the predictive value of maternal characteristics, biomarkers, and ultrasound scans have been evaluated for IUGR, too, but none have shown to be sufficiently clinically valuable (3), leaving the detection rate of IUGR around 31% (10).

Recently, a pilot-study by our group reported that increased second trimester values of salivary uric acid (sUA) were associated with later development of PE and pregnancy-induced hypertension (PIH) (11). Uric acid production is increased by hypoxia and metabolic stress (12), and though the pathophysiology of PE and IUGR remains unclear (3, 6), placental and endothelial dysfunction, including hypoxia and metabolic stress, are widely accepted as key factors (6, 13, 14). Further, sUA has shown to be superior to serum uric acid for detecting metabolic stress (15), perhaps partly due to a redirection of the uric acid metabolism toward the enteral route (16). Therefore, sUA is suggested as a far more sensitive biomarker of metabolic stress in pregnancy compared with serum uric acid, since serum uric acid has not shown potential as a predictive biomarker of PE (17).

This aligns with findings in the literature that in general, salivary biomarkers might be more sensitive to detect inflammation compared to serum biomarkers (18).

Furthermore, a recent publication by our research team found that sUA levels were significantly increased in pregnancies complicated by IUGR, suggesting that increased levels of sUA reflected placental dysfunction (19).

For this study, a novel smartphone-based self-test device was developed (Morgan Innovation and Technology, Petersfield, UK) (20) for weekly monitoring of sUA levels. The self-test was based on digital images of colorimetric test-strips, since these have shown to be able to monitor changes in sUA levels (21).

This study aimed to investigate the efficacy of this smartphone-based self-test, using colorimetric test-strips to measure sUA, to predict PE, PIH, and IUGR from 20 weeks gestation.

## Material and methods

### Design and study population

In total, 563 women attending their routine anomaly scan at gestational week 20–21 at Zealand University Hospital, Roskilde, Denmark, between August 2017 and July 2018 were included in this prospective cohort study. Prior to the scan, written study information was sent electronically to prospective study participants and oral information was given during their scan appointment. Participants were enrolled with an ID number, they were given a box with 20 test kits, provided with a personal QR-code, and instructed how to perform the sample collection using a smartphone-app. Inclusion criteria were age ≥ 18 years, good communicative skills in Danish or English, and having a smartphone. Exclusion criteria were fetal malformations detected on the ultrasound scan.

### Sample collection

**FIGURE 1:**
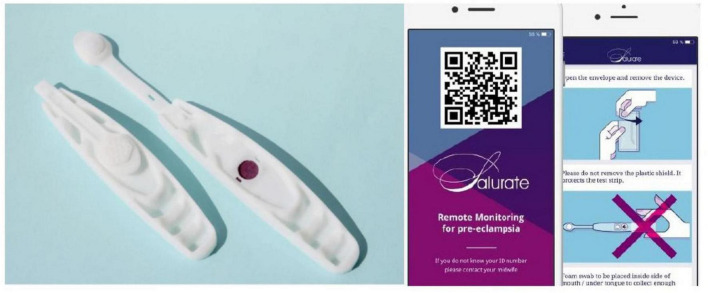
The self-test device with colorimetric test-strip and the assisting smartphone-app. Images reproduced with permission from the company Morgan Innovation and Technology.

The test-device used (Figure 1) is a plastic device, consisting of a buccal swab and a colorimetric test-strip, impregnated with a chemical solution including copper(II) and sodium bicinchoninate. Uric acid reduces Cu(II) to Cu(I), which reacts with sodium bicinchoninate, forming a purple chelate, which changes the test-strip color from white to purple (21).

The app guided women through the procedure of testing (Figure 1). First, the swab was pressed onto the tongue for 30 s and then on the test-strip. The test-device was thereafter placed on the box as a background, and a photograph including the test-strip, the ID number and a color reference scale was taken with their smartphone camera. After electronic approval of the photograph quality, the app sent it to a secure cloud database.

Women were asked to test weekly, first thing in the morning before brushing teeth, exercising, drinking, eating or smoking. The app sent a weekly reminder.

Self-reported baseline characteristics were collected at recruitment. Self-reported diagnoses of former PE or PIH were crosschecked in the records.

### Color analyses

The majority of smartphone cameras create color images based on the red green blue (RGB) color model, describing the color intensity of each pixel as a value between 0 and 255—the darker the color, the lower the value (22). However, other color models (the HSV, the YCbCr and the CIELAB models), derived from the RGB model have shown to provide greater data resolution (23, 24). Since no gold standard exists as to which color variables should be utilized for a colorimetric analysis (21, 25, 26), a methodological decision was made to assess and compare all individual variables derived from the four dominant color models in this study. We used Matlab (MathWorks Inc., Massachusetts, USA) to convert the shade of purple on each test-strip image into 12 color variables from (1) the RGB color model and (2) the HSV, YCrCr and CIELAB color models. All color models were utilized to provide the prediction of the different outcomes (PE, PIH, and IUGR) and six summary statistics (minimum, maximum, mean, standard deviation, range, median) were obtained for each of the color variables. This resulted in every image being described by 72 variables.

### Outcome measures

Main outcome measures were diagnosis of PE and PIH as classified by the International Society for the Study of Hypertension in Pregnancy (5), and ultrasound-based diagnosis of IUGR as defined by the International Federation of Gynecology and Obstetrics (3). Information on pregnancy outcomes were obtained from the electronical patient record after delivery, including gestational age (GA) at delivery, birthweight, measurements of elevated blood pressure and proteinuria, laboratory values, and diagnoses of PIH, PE, and IUGR. The records of all women with measurements of elevated blood pressure, diagnosis of PE or PIH were examined to validate the diagnoses of PE or PIH. In all women with either a diagnosis of IUGR in the electronical patient record or who delivered a newborn with a birthweight below the 10th percentile, the diagnosis of IUGR was validated by two members of the research team assessing the obstetric records and the ultrasound scans performed through the pregnancy from the software program Astraia (27) (GMBH, Munich, Germany). This was in order to correctly categorize IUGR vs. small-for-gestational-age newborns, since we in this study evaluated the outcome of IUGR and not small-for-gestational-age.

In addition, related adverse maternal and fetal outcome measures were obtained: diagnosis of eclampsia, diagnosis of hemolysis, elevated liver enzymes and low platelet count syndrome, antihypertensive treatment, anticonvulsive treatment, placental abruption, admission to neonatal intensive care unit, respiratory distress syndrome, fetal death, neonatal death (< 28 days postpartum) and maternal death.

### Sample size calculation and statistical analyses

Given the novelty of this approach, no previous data were available to inform a power calculation. A methodological decision was made of basing the sample-size estimation on the assumption that colorimetric data from 40 women with PE would be sufficient in order to assess the discriminatory ability of the individual color variables. The sample size calculation was based on an assumed 10% prevalence of PE and an expected compliance rate at 80%, resulting in an aim to include 500 women. The compliance rate of a participant was calculated as number of weeks with a submitted test divided by the total number of weeks from recruitment to delivery, as a percentage.

**FIGURE 2:**
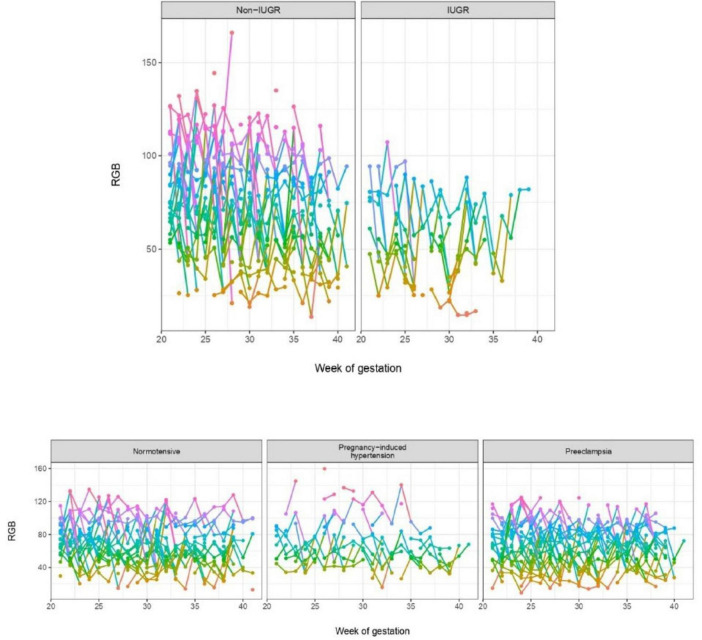
Weekly minimum RGB values through the pregnancy from 20 to 21 weeks of gestation to delivery, for pregnancies showing normal growth vs. intrauterine growth restriction and for normotensive pregnancies, pregnancy-induced hypertension and pre-eclampsia, respectively. A random subset of women was chosen for visualization for normotensive, pregnancy-induced hypertension, pre-eclampsia and pregnancies unaffected by IUGR, while all IUGR cases are visualized. On the panels, the color of the lines reflect the RGB at the *y*-axis from high to low, with purple, blue, green and orange, respectively.

The longitudinal pattern of the RGB values were visualized in Figure 2 according to outcomes of PE, PIH and IUGR, as a part of the exploratory data analysis. The lowest RGB value for each test, representing the part of the test-strip most saturated with saliva, was chosen for analysis. Only women who had submitted a test each week were included.

**FIGURE 3:**
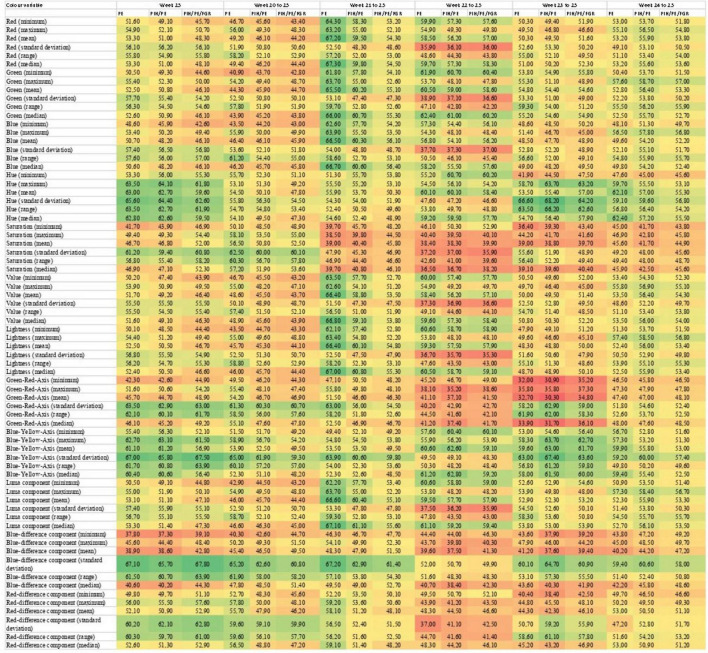
Heat map of the area under the curve (AUC) for each individual color variable for the outcomes of PE, PE and/or PIH, and PE and/or PIH, and/or IUGR, at 25 weeks of gestation and for the weekly color change between 20 and 25 weeks of gestation, respectively. The AUC is a value between zero and 100 and the colors of the heat map visualize the individual values. Green is closer to 100 and red is closer to zero. The color variables are derived from the four dominant color spaces. R, red; G, green; B, blue; H, Hue; S, saturation; V, value; Y, luma component; Cb, blue-difference component; Cr, red-difference component; L, lightness; A, green-red-axis; B, blue-yellow-axis.

The discriminatory value of the 72 individual color variables was assessed using the area under the receiver operating characteristic curve (AUC), reported as a value between zero and 100 with one decimal and 95% CI, not accounting for multiple testing. The benchmark lower limit for AUC is 50 which can be achieved without performing the colorimetric tests. Therefore, the discriminatory ability is based on a value of the AUC between 50 and 100, the higher the AUC, the better the discriminatory ability. We assessed the discriminatory ability of each of the 72 color variables at week 25 of gestation for events of PE, PE/PIH combined and PE/PIH/IUGR combined occurring after gestational week 25, only including participants that were event-free at week 25. In addition, the discriminatory ability of the weekly color change between 20 and 25 weeks of gestations were evaluated. Women, who had submitted a test in these weeks, were included for analyses regardless of missing values in other weeks. The results are presented in a heat map (Figure 3). Data analysis was performed using R software (version 4.0.3 for Windows; R Development Core Team, 2020) (28).

### Ethics statement

The study was approved the Regional Committee on Health Research Ethics (SJ-583), the Danish Data Protection Agency (REG-140-2018) and the Danish Medicines Agency (2016100924), and informed, written consent was obtained at enrolment.

## Results

In total, 563 women were enrolled of which 68 women were excluded because of poor test-paper quality in the first batch, leaving 495 women eligible for analyses. The characteristics of the study population at delivery are summarized in Table 1 with categorical variables presented as frequencies and continuous variables presented as median values with interquartile ranges.

**TABLE 1:** Characteristics of the participants, *N* = 495.

Characteristics		Normotensive, non-IUGR pregnancies, *n* = 438	PE and/or PIH and/or IUGR, *n* = 56	PE, *n* = 34	PIH, *n* = 17	PE and/or PIH, *n* = 51	IUGR, *n* = 10
**Age [year]**							
	19–24	50 (11.4)	7 (12.5)	5 (14.7)	2 (11.8)	7 (13.7)	0 (0.0)
	25–29	139 (31.7)	13 (23.2)	6 (17.6)	7 (41.2)	13 (25.5)	1 (10.0)
	30–34	151 (34.5)	20 (35.7)	12 (35.3)	5 (29.4)	17 (33.3)	4 (40.0)
	35–39	81 (18.5)	12 (21.4)	9 (26.5)	1 (5.9)	10 (19.6)	4 (40.0)
	40–44	15 (3.4)	4 (7.1)	2 (5.9)	2 (11.8)	4 (7.8)	1 (10.0)
	45–50	2 (0.5)	0 (0.0)	0 (0.0)	0 (0.0)	0 (0.0)	0 (0.0)
**BMI [kg/m^2^]**		Information missing: 3 (0.7)					
	< 18.5	12 (2.7)	1 (1.8)	0 (0.0)	0 (0.0)	0 (0.0)	1 (10.0)
	18.5–24.9	247 (56.4)	22 (39.3)	13 (38.2)	6 (35.3)	19 (37.3)	5 (50.0)
	25.0–29.9	108 (24.7)	14 (25.0)	9 (26.5)	4 (23.5)	13 (25.5)	3 (30.0)
	> 29.9	68 (15.5)	19 (33.9)	12 (35.3)	7 (41.2)	19 (37.3)	1 (10.0)
Current smoker		29 (6.7)	4 (7.1)	0 (0.0)	2 (11.8)	2 (3.9)	2 (20.0)
**Parity**							
	Nullipara	182 (41.8)	28 (50.0)	20 (58.8)	6 (35.3)	26 (51.0)	7 (70.0)
	Multipara	253 (58.1)	28 (50.0)	14 (41.2)	11 (64.7)	25 (49.0)	3 (30.0)
**Number of fetuses**							
	Singleton	431 (99.1)	55 (98.2)	34 (100)	17 (100)	51 (100)	9 (90.0)
	Gemelli	4 (0.9)	1 (1.8)	0 (0.0)	0 (0.0)	0 (0.0)	1 (10.0)
**History of pre-eclampsia**		6 (1.4)	6 (10.7)	5 (14.7)	1 (5.9)	6 (11.8)	0 (0.0)
**Birthweight [grams]**		Information missing: 35 (8.0)	Information missing: 1 (1.8)				Information missing: 1 (10.0)
	< 2,500	10 (2.3)	8 (14.3)	4 (11.8)	1 (5.9)	5 (9.8)	6 (60.0)
	2,500–3,499	155 (35.4)	25 (44.6)	16 (47.1)	8 (47.1)	24 (47.1)	3 (30.0)
	3,500–4,500	224 (51.1)	22 (39.3)	14 (41.2)	8 (47.1)	22 (43.1)	0 (0.0)
	> 4,500	14 (3.2)	0 (0.0)	0 (0.0)	0 (0.0)	0 (0.0)	0 (0.0)
**Gestational age at delivery [days] (weeks)**		Information missing: 17 (3.9)					
	< 224 (< 32 weeks)	3 (0.7)	3 (5.4)	1 (2.9)	0 (0.0)	1 (2.0)	3 (30.0)
	224–237 (32–34 weeks)	2 (0.5)	1 (1.8)	0 (0.0)	0 (0.0)	0 (0.0)	1 (10.0)
	238–258 (34–37 weeks)	17 (3.9)	3 (5.4)	1 (2.9)	1 (5.9)	2 (3.9)	2 (20.0)
	259–279 (37–40 weeks)	150 (34.2)	28 (50.0)	24 (70.6)	8 (47.1)	32 (62.7)	4 (40.0)
	> 279 (> 40 weeks)	249 (56.8)	21 (37.5)	8 (23.5)	8 (47.1)	16 (31.4)	0 (0.0)
**Compliance**		Information missing: 4 (0.9)					
	Samples submitted	16 (14)	16 (13)	18 (12)	11 (15)	17 (13)	12 (4)
	Compliance rate (%)	85.8 (75)	94.3 (64.3)	100 (50)	73.3 (78.9)	94.4 (67.4)	96.7 (8)

Frequencies are *n* (%) or median (interquartile range). IUGR, intrauterine growth restriction; PE, pre-eclampsia; PIH, pregnancy-induced hypertension; BMI, body mass index; GA, gestational age.

The mean compliance rate was 67% (0–100%). After 308 women were enrolled, the preliminary mean compliance rate was 56%, leading to an update of the smartphone-app. Subsequently, the mean compliance rate increased to 77%.

Seventeen (3.4%) women developed PIH without progression to PE, 10 (2.0%) developed IUGR, and 34 (6.9%) developed PE, of which five (14.7%) had concomitant IUGR, two (5.9%) developed early-onset PE (< 34 + 0 weeks of gestation), and eight (23.5%) developed preterm PE (< 37 + 0 weeks of gestation). Five (14.7%) women with PE received intravenous antihypertensive treatment and one (2.9%) received Magnesium Sulfate, too. The incidence of main outcomes measures and adverse maternal and fetal outcomes are reported in Table 2.

**TABLE 2:** Main outcomes measures and adverse maternal and fetal outcomes.

Outcomes within the study population, *N* = 495 women	*n* (%)
Overall PE	34 (6.9)
Severe hypertension (blood pressure: systolic ≥ 160 and/or diastolic ≥ 110 mm Hg)	15 (44.1)
Early-onset PE < 34 weeks of gestation	2 (5.9)
Preterm PE < 37 weeks of gestation	8 (23.5)
Term PE > 37 weeks of gestation	26 (67.6)
Antihypertensive treatment due to PE	23 (67.6)
Oral	23 (100.0)
Intravenous	5 (14.7)
Treatment with MgSO4	1 (2.9)
PIH	17 (3.4)
Antihypertensive treatment due to PIH	4 (23.5)
Oral	4 (100.0)
Intravenous	0 (0.0)
IUGR	10 (2.0)
Placental abruption related to PE	1 (2.9)
Preterm delivery for any reason (< 37 weeks of gestation)	29 (5.9)
Preterm delivery associated with PE, PIH or IUGR	7 (1.4)
Admission to NICU related to PIH/PE/IUGR	10 (2.0)
Respiratory distress syndrome related to PIH/PE/IUGR	1 (0.2)
Eclampsia	0 (0)
Hemolysis, elevated liver enzymes, low platelets syndrome	0 (0)
Fetal death/neonatal death/maternal death	0 (0)

Frequencies are reported as *n* (%).

For all women, the longitudinal pattern of RGB values fluctuated throughout the pregnancy with a wide within-individual variation between the weeks, as shown in Figure 2, and discrimination was not observed for any of the three outcomes. For IUGR, the RGB values ranged from 16 to 107 (out of a possible range of 0–255), with only one sample exceeding 100. In contrast to this, the range of RGB values was wider (4 to 177) in women without IUGR, with multiple measurements above 100.

The AUC’s for the 72 individual color variables regarding the three outcomes and six time points, found that 8.2% of the analyses showed significant discriminatory ability (Figure 3), defined by the AUC exceeding the benchmark lower limit for AUC at 50, with the top five variables AUC’s ranging from 55.6 (43.5–67.7) to 68.2 (59.2–77.2). Of these, 29 (27.4%) were summaries of the Hue color variable from the HSV color model. However, discriminatory ability was not consistent regarding gestational age or outcome group as shown in Figure 3.

## Discussion

### Main findings

To our knowledge, this prospective cohort study in 495 pregnant women is the first of its kind to evaluate the clinical efficacy of applying a digital, salivary colorimetric self-test for the prediction of PE, PIG, and IUGR from mid-pregnancy.

The observed compliance rate indicates that the pregnant women found weekly, salivary self-testing to be an acceptable and feasible solution. This is in alignment with a recent qualitative publication by our group that found that pregnant women have a positive attitude toward self-tests for PE (29). The discriminatory ability of neither the individual color variables nor combined RGB values were sufficient for clinical application at this stage. However, a visual difference was observed regarding the absence or presence of IUGR.

### Interpretation of results

The assessment of the discriminatory ability of the 72 color variables found that 8.2% of the analyses showed discriminatory ability, but overall, individual color analysis was not found to have predictive value for the evaluated outcomes. There might be different reasons for this. There is growing evidence that PE is comprised by different phenotypes of maternal cardiovascular dysfunction or placental dysfunction (6). In the study population, the 34 women with an event of PE differed concerning the severity of the disease, the time of onset, the level of treatment needed and whether fetal growth was affected, with the majority of the women not suffering from severe PE. Therefore, the differences within the study population of women with PE could have weakened the results. Alternatively, methodological limitations as lack of statistical power and too wide variation within the colorimetric test-strips to detect small changes in sUA could have affected the results negatively. However, the analyses allowed for comparison of individual color variables derived from the colorimetric test-strip, finding that the Hue variable accounted for 27.4% of the variables with discriminatory ability. Given the lack of a golden standard for colorimetric analysis of digital images, this study adds information to the rapidly growing field of smartphone-assisted colorimetric test-strips (25, 26, 30). The observed RGB values showed a wide within-individual variation, suggesting limited ability for identification of women at risk of hypertensive disorders of pregnancy. This phenomenon is familiar from studies on blood glucose which demonstrate wide within-individual variation in measurements compared to measurements of HbA1C (31). Nevertheless, the observation that women without IUGR had multiple RGB values peaking above 100 (reflecting a lighter shade of purple and lower levels of sUA) while women with IUGR had continuously low values (reflecting darker shades of purple and higher levels of sUA), indicates a potential clinical utility for the test and supports the hypothesis that placental pathology causes hypoxia and metabolic stress, resulting in increased levels of sUA (12). However, since the number of women with IUGR was low, it was not possible to quantify the discriminatory ability of the colorimetric test-strip for IUGR as a sole outcome in this cohort. Further, in women without IUGR, the RGB values fluctuated to low values, too. This is in line with our previously reported finding that in normal pregnancies sUA levels fluctuate on a weekly basis (11). Further, a recently published study showed that in low-risk, pregnant women, the variation of sUA were greater in the morning compared to mid-day and evening (32). Therefore, testing first thing in the morning might have contributed to an increased variation of sUA values in women without an outcome. In addition, it has been shown that sUA levels are sensitive to physical activity and diurnal variations (15), and therefore even minor deviations from the sampling procedure could possibly increase sUA levels. Therefore, this should be taken into consideration in future research.

### Strengths and limitations

The major strengths of the study include the prospective cohort design, close follow up and high degree of mean compliance balanced in all subgroups. In addition, the procedure of testing first thing in the morning eliminated known influential factors on the levels of sUA such as diurnal changes, physical activity (15) and possible dilution of the uric acid concentrations by consumption of food or beverage. Another strength was the careful validation of diagnoses, avoiding misclassification of events (33). Further, focusing the analyses on the gestational time prior to diagnoses and thereby evaluating the predictive instead of the diagnostic potential of the test-strip was a strength, aligning with clinical needs (34). Week 25 was chosen, since it is a time of clinical importance as sonographic monitoring of fetal growth in women with high risk of IUGR is recommended to start in week 24–25 (3).

The study also has a number of limitations. Firstly, the discriminatory value of the colorimetric test-strips was assessed for each of the 72 color variables individually, which did not allow for assessment of the combination of color variables in the four respective color models. Another limitation to this study was that the standard deviation or the correlation coefficient between the laboratory values of sUA and the color values of the digital images were not available before the study started. Therefore, unknown variations in the colorimetric test-strip might have affected the findings negatively. A further limitation was that the prevalence of diagnoses and the mean compliance rate before updating the app were lower than expected, decreasing the power of the study, possibly resulting in a type 1 error. In addition, due to missing values, different women were investigated in the different weeks, making it difficult to compare groups. Also, the discriminatory analysis did not adjust for multiple testing, why the findings of some individual color variables being significant according to their AUC might be change findings. Further, the analyses were not adjusted for possible influence by medical diseases, since this information was unavailable. Finally, it is a limitation to the study, that information on oral health in the participants was unavailable, since animal models suggest that gingival modifications through pregnancy can affect inflammatory biomarkers (35).

### Clinical implications

This study examined a novel, digital health solution for major pregnancy complications of PE, PIH, and IUGR. The high compliance rate observed in the study, holds clinical importance, given the growing global demand for telemedical solutions (36). The concept of digital, salivary monitoring has potential to be a major global health resource, especially in in rural areas with long travel time to primary care centers as even relatively deprived communities will often have good access to mobile phone technologies (37). While this study did not find that the tested iteration of the self-test evaluated had clinical value for the prediction of PE, PIH, or IUGR, the technology regarding smartphone-assisted colorimetric test-strips are rapidly evolving, with refinements of the technique being published after the conduct of this study (25, 26, 30, 38). The observed difference regarding the RGB values for IUGR suggests that a further development of the technology might have potential to rule out a risk of IUGR. Potentially, this would not only spare women time from attending growth scans every second week, but also avoid maternal worry related to unnecessary fetal monitoring (39).

A recent qualitative publication by our group showed that pregnant women found the concept of self-testing easy, attractive and empowering. However, the study also found that self-testing should be combined with appropriate professional assistance regarding the importance of reacting to subjective changes in bodily sensations, including fetal movements (29).

## Conclusion

This study found that weekly, digital self-test for the prediction of PE, PIH, and IUGR had a high compliance rate among pregnant women and could be an acceptable solution. Individual color analysis of the four dominant color models found that 8.2% had discriminatory ability with the Hue color variable being superior, adding important insights to the evolving field of smartphone-assisted colorimetric self-tests. However, the iteration of the test-device applied in this study was not found to have sufficient predictive value to be clinically useful. Observing the combined RGB color value suggested that sUA measurement might be more useful for a subgroup of women with placental dysfunction, instead of overall PE. However, it was beyond the scope of this study, to validate this finding further. Further research should seek to further confirm the validity of sUA and to explore the predictive value of combined color analysis in larger cohorts of women with placental disorders.

## Data Availability

The raw data supporting the conclusions of this article will be made available by the authors, without undue reservation.

## References

[1] Steegers EAP von Dadelszen P Duvekot JJ Pijnenborg R. Pre-eclampsia. *Lancet.* (2010) 376:631–44. 10.1016/S0140-6736(10)60279-6 20598363

[2] Romo A Carceller R Tobajas J. Intrauterine growth retardation (IUGR): epidemiology and etiology. *Pediatric Endocrinol Rev.* (2009) 6:5. 19404231

[3] Melamed N Baschat A Yinon Y Athanasiadis A Mecacci F Figueras F. FIGO (International Federation of Gynecology and Obstetrics) initiative on fetal growth: best practice advice for screening, diagnosis, and management of fetal growth restriction. *Int J Gynaecol Obstet.* (2021) 152(Suppl. 1):3–57. 10.1002/ijgo.13522 33740264 PMC8252743

[4] Stevens W Shih T Incerti D Ton TGN Lee HC Peneva D. Short-term costs of preeclampsia to the United States health care system. *Am J Obstet Gynecol.* (2017) 217:237–48.e16. 10.1016/j.ajog.2017.04.032 28708975

[5] Brown MA Magee LA Kenny LC Karumanchi SA McCarthy FP Saito S. Hypertensive disorders of pregnancy: ISSHP classification, diagnosis, and management recommendations for international practice. *Hypertension.* (2018) 72:24–43. 10.1161/HYPERTENSIONAHA.117.10803 29899139

[6] Myatt L Roberts JM. Preeclampsia: syndrome or disease? *Curr Hypertens Rep.* (2015) 17:83. 10.1007/s11906-015-0595-4 26362531

[7] Poon LC Shennan A Hyett JA Kapur A Hadar E Divakar H. The International Federation of Gynecology and Obstetrics (FIGO) initiative on pre-eclampsia: a pragmatic guide for first-trimester screening and prevention. *Int J Gynecol Obstetr.* (2019) 145:1–33. 10.1002/ijgo.12802 31111484 PMC6944283

[8] Tan MY Wright D Syngelaki A Akolekar R Cicero S Janga D. Comparison of diagnostic accuracy of early screening for pre-eclampsia by NICE guidelines and a method combining maternal factors and biomarkers: results of SPREE. *Ultrasound Obstetr Gynecol.* (2018) 51:743–50. 10.1002/uog.19039 29536574

[9] Rolnik DL Wright D Poon LC O’Gorman N Syngelaki A de Paco Matallana C. Aspirin versus Placebo in pregnancies at high risk for preterm preeclampsia. *N Engl J Med* (2017) 377:613–22. 10.1056/NEJMoa1704559 28657417

[10] Andreasen LA Tabor A Nørgaard LN Taksøe-Vester CA Krebs L Jørgensen FS. Why we succeed and fail in detecting fetal growth restriction: A population-based study. *Acta Obstetr Gynecol Scand.* (2021) 100:893–9. 10.1111/aogs.14048 33220065

[11] Püschl IC Bonde L Reading IC Maguire P Macklon NS Van Rijn BB. Salivary uric acid as a predictive test of preeclampsia, pregnancy-induced hypertension and preterm delivery: A pilot study. *Acta Obstet Gynecol Scand.* (2020) 99:1339–45. 10.1111/aogs.13888 32350850

[12] Chung HY Baek BS Song SH Kim MS Huh JI Shim KH. Xanthine dehydrogenase/xanthine oxidase and oxidative stress. *Age (Omaha).* (1997) 20:127–40. 10.1007/s11357-997-0012-2 23604305 PMC3455892

[13] Sikkema JM van Rijn BB Franx A Bruinse HW de Roos R Stroes ES. Placental superoxide is increased in pre-eclampsia. *Placenta.* (2001) 22:304–8. 10.1053/plac.2001.0629 11286565

[14] Burton GJ Jauniaux E. Pathophysiology of placental-derived fetal growth restriction. *Am J Obstet Gynecol.* (2018) 218:S745–61. 10.1016/j.ajog.2017.11.577 29422210

[15] Owen-Smith B Quiney J Read J. Salivary urate in gout, exercise, and diurnal variation. *Lancet.* (1998) 351:1932. 10.1016/S0140-6736(05)78616-5 9654268

[16] Sorensen LB. Role of the intestinal tract in the elimination of uric acid. *Arthritis Rheum.* (1965) 8:694–706. 10.1002/art.1780080429 5859543

[17] Corominas AI Medina Y Balconi S Casale R Farina M Martínez N Assessing the role of uric acid as a predictor of preeclampsia. *Front Physiol.* (2022) 12:785219. 10.3389/fphys.2021.785219 35095555 PMC8794766

[18] Riis JL Bryce CI Ha T Hand T Stebbins JL Matin M Adiponectin: serum-saliva associations and relations with oral and systemic markers of inflammation. *Peptides.* (2017) 91:58–64. 10.1016/j.peptides.2017.03.006 28363793

[19] Püschl IC Thaneswaran Vyramuthu M Bonde L Lebech M Iraqi Møller H Vauvert F. Is salivary uric acid, a putative biomarker of pre-eclampsia, of maternal, placental, or fetal origin? *Eur J Obstet Gynecol Reprod Biol.* (2024) 295:34–41. 10.1016/j.ejogrb.2024.02.003 38330864

[20] SecretSource. *Morgan Innovation and Technology.* (2020). Available online at: https://morgan-iat.co.uk/projects/salurate/ (accessed June 13, 2020).

[21] Blicharz TM Rissin DM Bowden M Hayman RB DiCesare C Bhatia JS. Use of colorimetric test strips for monitoring the effect of hemodialysis on salivary nitrite and uric acid in patients with end-stage renal disease: a proof of principle. *Clin Chem.* (2008) 54:1473–80. 10.1373/clinchem.2008.105320 18676588 PMC2710817

[22] Hunt RWG. *The Reproduction of Colour.* 6th ed. Chichester: Wiley–IS&T Series in Imaging Science and Technology (2004). 10.1002/0470024275

[23] Shi RB Qiu J Maida V. Towards algorithm-enabled home wound monitoring with smartphone photography: A hue-saturation-value colour space thresholding technique for wound content tracking. *Int Wound J.* (2019) 16:211–8. 10.1111/iwj.13011 30379398 PMC7948822

[24] Rook A John MM Post A Razavi M. Alcohol ablation of cardiac tissues quantified and evaluated using CIELAB euclidean distances. *Tex Heart Inst J.* (2020) 47:265–70. 10.14503/THIJ-19-7140 33472218 PMC7819450

[25] Hou P Deng R Guo J Chen W Li X Yu HZ. A WiFi scanner in conjunction with disposable multiplex paper assay for the quantitation of disease markers in blood plasma. *Anal Bioanal Chem.* (2021) 413:4625–34. 10.1007/s00216-021-03234-6 33661349

[26] Fan K Zeng J Yang C Wang G Lian K Zhou X. Digital quantification method for sensitive point-of-care detection of salivary uric acid using smartphone-assisted μPADs. *ACS Sens.* (2022) 7:2049–57. 10.1021/acssensors.2c00854 35820152

[27] nexus-astraia. *Home - Astraia [Internet].* (2022). Available online at: https://www.nexus-astraia.com/ (accessed June 14, 2022).

[28] R Core Team. *R: The R Project for Statistical Computing [Internet].* (2021). Available online at: https://www.r-project.org/ (accessed June 22, 2021).

[29] Püschl IC de Wolff MG Broberg L Macklon N Hegaard HK. Pregnant women’s attitudes to and experiences with a smartphone-based self-test for prediction of pre-eclampsia: a qualitative descriptive study. *BMJ Open.* (2023) 13:e065575. 10.1136/bmjopen-2022-065575 37221028 PMC10230945

[30] Li NS Chen YT Hsu YP Pang HH Huang CY Shiue YL. Mobile healthcare system based on the combination of a lateral flow pad and smartphone for rapid detection of uric acid in whole blood. *Biosens Bioelectron.* (2020) 164:112309. 10.1016/j.bios.2020.112309 32479340

[31] Carlsen S Petersen PH Skeie S Skadberg Ø Sandberg S. Within-subject biological variation of glucose and HbA(1c) in healthy persons and in type 1 diabetes patients. *Clin Chem Lab Med.* (2011) 49:1501–7. 10.1515/CCLM.2011.233 21631391

[32] Riis JL Cook SH Letourneau N Campbell T Granger DA Giesbrecht GF. Characterizing and evaluating diurnal salivary uric acid across pregnancy among healthy women. *Front Endocrinol.* (2022) 13:813564. 10.3389/fendo.2022.813564 35370953 PMC8971544

[33] Klemmensen AK Olsen SF Osterdal ML Tabor A. Validity of preeclampsia-related diagnoses recorded in a national hospital registry and in a postpartum interview of the women. *Am J Epidemiol.* (2007) 166:117–24. 10.1093/aje/kwm139 17556761

[34] Myatt L. The prediction of preeclampsia: the way forward. *Am J Obstet Gynecol.* (2020) 226:S1102–7.e8. 10.1016/j.ajog.2020.10.047 33785181

[35] Contreras-Aguilar MD López-Arjona M Martínez-Miró S Escribano D Hernández-Ruipérez F Cerón JJ Changes in saliva analytes during pregnancy, farrowing and lactation in sows: A sialochemistry approach. *Vet J.* (2021) 273:105679. 10.1016/j.tvjl.2021.105679 34148602

[36] Labrique A Agarwal S Tamrat T Mehl G. WHO Digital Health Guidelines: a milestone for global health. *NPJ Digit Med.* (2020) 3:120. 10.1038/s41746-020-00330-2 33015373 PMC7501250

[37] WHO. *Be Healthy Be Mobile [Internet].* (2022). Available online at: https://www.who.int/initiatives/behealthy (accessed May 30, 2022).

[38] Chen X Chen J Wang F Xiang X Luo M Ji X. Determination of glucose and uric acid with bienzyme colorimetry on microfluidic paper-based analysis devices. *Biosens Bioelectron.* (2012) 35:363–8. 10.1016/j.bios.2012.03.018 22472530

[39] Harris JM Franck L Michie S. Assessing the psychological effects of prenatal screening tests for maternal and foetal conditions: a systematic review. *J Reprod Infant Psychol.* (2012) 30:222–46. 10.1080/02646838.2012.710834

